# An Improved Detecting Algorithm of Moving Targets for Airborne Maritime Surveillance Radar

**DOI:** 10.3390/s25020560

**Published:** 2025-01-19

**Authors:** Zhefeng Wu, Gang Zhang, Xinchun Wang, Peng Sun, Yanchao Lin

**Affiliations:** Beijing Aerospace Automatic Control Institute, Beijing 100854, China; wuzheming001@126.com (Z.W.); dr.gang@foxmail.com (G.Z.); sunpeng7315@126.com (P.S.); linyanchao123@sina.com (Y.L.)

**Keywords:** target tracking, envelope alignment, phase filtering, maritime surveillance radar

## Abstract

The traditional method is capable of detecting and tracking stationary and slow-moving targets in a sea surface environment. However, the signal focusing capability of such a method could be greatly reduced especially for those variable-speed targets. To solve this problem, a novel tracking algorithm combining range envelope alignment and azimuth phase filtering is proposed. In this method, the motion of the airborne maritime surveillance radar platform is firstly compensated for target echoes. Secondly, range envelope alignment is performed to correct the unpredictable range migration of the target after pulse compression. The higher-order phase difference between the adjacent pulses is estimated and compensated. Ultimately, such pulse series are accumulated through azimuth Fourier transform. Traditional methods compensate only for platform motion, limiting their ability to handle variable-speed targets. The proposed algorithm addresses this limitation by compensating for both platform and target motion, significantly improving signal focusing and tracking accuracy. A detailed analysis shows that our algorithm can significantly increase the signal accumulating gain and improve the focusing effect. The simulation results are provided to demonstrate the effectiveness of the proposed algorithm.

## 1. Introduction

The airborne maritime surveillance radar, as a key requirement for ocean surface monitoring, can detect and track sea surface targets in long-range, wide-range, and multi-angle [1,2] situations. Provisions of those radars are, therefore, essential and gaining an increasing interest in the remote sensing scientific community [3].

The airborne maritime surveillance radar is capable of sorting maritime targets simultaneously over the horizon. It obtains the target position and velocity information through continuous-time coverage and Doppler estimation [4]. At present, the airborne maritime surveillance radar has been widely applied in maritime traffic control, fisheries management, maritime search and rescue, and illegal immigration and smuggling control [5]. Due to the practical value and urgent demands of the airborne maritime surveillance radar, several working systems have been deployed in the past several years. The American SeaVue radar, as an X-band radar installed on fixed-wing aircraft, can achieve maritime monitoring, search, and rescue in sea surface environment [5]. The French Ocean radar with a detection range of 120 km has been in continuous operation near Brest since 2006 [6]. Israel’s EL/M-2022A/H/U radar with a power consumption of 3.3 kW can detect tiny sea surface targets within 200 nm [4,5,6]. The seaspray 5000E and 7500E, as an active electronically scanned array multi-mode surveillance radar, can achieve long-range search and exceptional small target detection [7,8]. All these systems have a high measuring accuracy and can detect and track a variety of sea surface targets effectively for different purposes.

With the development of airborne maritime surveillance systems, a series of algorithms have been proposed to improve the tracking and detecting performance for sea surface targets. Yu et al. introduced a novel sea-clutter-suppressing method to obtain a better detecting result through adaptive dual-threshold sparse Fourier transform [9]. L. Yang et al. introduce a detector based on weighted information entropy, amplifying the difference between the target and its surroundings, to suppress sea clutter and the noise [10,11]. Weibo Huo et al. propose a ship-detecting method to distinguish the ship targets from the complex backgrounds of SAR [12]. To increase the detection probability for targets, Magraner et al. presented a cell averaging constant false alarm rate (CFAR) detection algorithm [13]. In [14], a convolutional neural network (CNN) and metric learning are combined to achieve sea surface target classification and detection. Several studies have explored maritime radar target detection and tracking. For instance, Farid et al. proposed a modified A-Star approach for path planning in obstacle-cluttered environments [15]. Marino et al. presented a 3D localization and tracking method for multiplatform radar networks [16]. These works provide valuable context for our proposed algorithm.

Those works mainly focus on sea clutter suppression and effective signal discovery [9,10,11,12,13,14,15,16,17]. However, the impact of target motion on radar echo processing is not considered. On the one hand, the unpredictable speed of the target results in a serious performance degradation for envelope alignment. Such range migration exceeding a pixel limit will seriously affect the signal focusing. On the other hand, target motion induces a higher-order phase error along the azimuth direction and decreases the signal accumulating gain seriously. To solve those problems, a novel tracking algorithm combining range envelope alignment and azimuth phase filtering is proposed. In this method, radar speed compensation and range envelope alignment are initially performed. At the second stage, the higher-order phase difference between the adjacent pulses is estimated and compensated. Ultimately, such pulse series are accumulated through azimuth Fourier transform [18].

The remainder of this paper is organized as follows. Section 2 presents the principle of the traditional tracking algorithm and the airborne maritime surveillance radar geometric configuration. In Section 3, the proposed range envelope alignment and azimuth phase filtering algorithm are introduced with a detailed tracking scheme. Experimental analyses of the proposed method are provided in Section 4 and conclusions are drawn in Section 5.

## 2. Principle of Traditional Tracking Algorithm of Maritime Moving Targets

Traditional tracking algorithms focus solely on compensating for platform motion to mitigate the relative motion effects in the received signals. This approach effectively reduces signal distortions caused by platform dynamics but fails to address the range migration and phase errors introduced by variable-speed targets, leading to significant signal defocusing and reduced tracking accuracy.

These algorithms typically assume a constant target velocity and rely on simplified motion models to align received signals. However, in dynamic maritime environments, where target velocities can vary unpredictably, such assumptions become invalid, resulting in severe performance degradation. Moreover, the inability to compensate for higher-order motion effects further limits the applicability of traditional methods to scenarios involving high-speed or variable-speed targets.

In this section, the model for the detecting and tracking targets scenario is first presented, followed by the signal model of the target echo and compressed signals. Subsequently, the traditional range migration is implemented through radar speed compensation. Meanwhile, the resultant signals are derived through pulse compression along the azimuth direction.

### 2.1. Geometric Configuration

**Figure 1:**
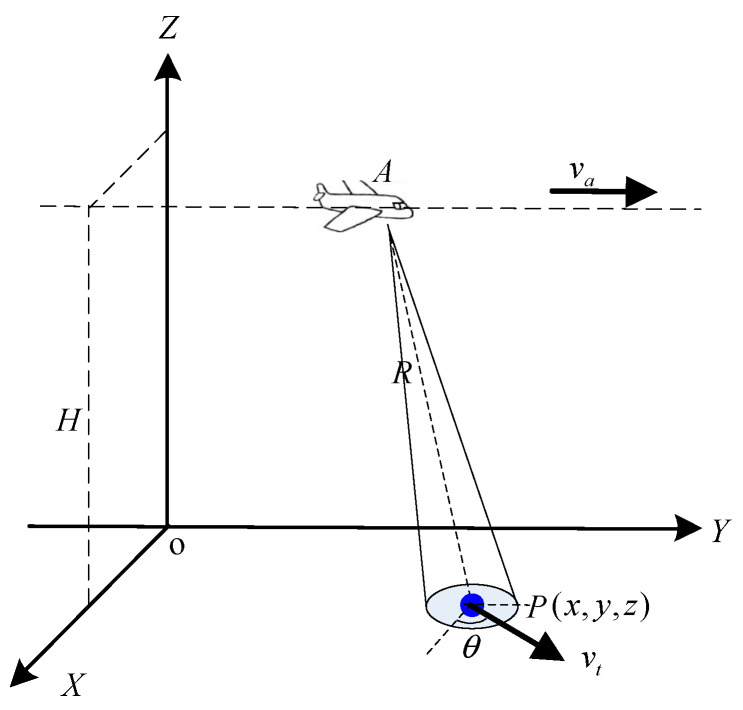
Airborne maritime surveillance radar geometry.

A schematic illustration for the target detecting and tracking considered in our work is shown in Figure 1. Point A represents the antenna of the airborne maritime surveillance radar. $\left[x_{0}+\left(v_{t}\cos\theta \right)t_{m}+\left(a_{t}\cos\theta \right){t_{m}}^{2},y_{0}+\left(v_{t}\sin\theta \right)t_{m}+\left(a_{t}\sin\theta \right){t_{m}}^{2},z_{0}\right]$ is the location of an arbitrary target P on the ground at slow time $t_{m}$. θ represents the angle between the speed of target and X-axis. Since the SAR platform flies along the y-axis at a fixed altitude with a speed of $v_{a}$, we suppose that the instantaneous positions of the radar antenna A is $\left(X_{s},v_{a}t_{m},Z_{s}\right)$ at slow time $t_{m}$. The distance between the antenna and the target P is represented by $R(t_{m})$. XOY (ground plane) denotes the target focus plane. The radar achieves sea surface target detection through periodic scanning and continuous tracking.

As can be seen, the instantaneous slant range between the radar and the sea surface target is calculated by the following:

(1) $$R\left(t_{m}\right)=\sqrt{{\left[X_{s}-x_{0}-\left(v_{t}\cos\theta \right)t_{m}-\left(a_{t}\cos\theta \right){t_{m}}^{2}\right]}^{2}+{\left[v_{a}t_{m}-y_{0}-\left(v_{t}\sin\theta \right)t_{m}-\left(a_{t}\sin\theta \right){t_{m}}^{2}\right]}^{2}+{\left(Z_{s}-z_{0}\right)}^{2}}$$

The range $R\left(t_{m}\right)$ can be expanded to a Taylor series about $t_{m}$ as

(2) $$R\left(t_{m}\right)=R_{0}-\left[\frac{\left(X_{s}-x_{0}\right)v_{t}\cos\theta -y_{0}\left(v_{t}\sin\theta -v_{a}\right)}{R_{0}}\right]t_{m}+o\left({t_{m}}^{2}\right)$$

where $o\left({t_{m}}^{2}\right)$ represents the higher-order term in the Taylor series expansion of $R\left(t_{m}\right)$, and

(3) $$R_{0}=\sqrt{{\left(X_{s}-x_{0}\right)}^{2}+{\left(y_{0}\right)}^{2}+{\left(Z_{s}\right)}^{2}}$$

These quantities in Equation (2) can readily be expressed in terms of radar and target parameters. $\left[\left(X_{s}-x_{0}\right),-y_{0},Z_{s}\right]$ and $\left[v_{t}\cos\theta ,\left(v_{t}\sin\theta -v_{a}\right),0\right]$, respectively, denote the vectors of beam steering and relative velocity between the target and radar. Ignoring the impact of the higher-order term $o\left({t_{m}}^{2}\right)$ on range envelope alignment and azimuth focusing, Formula (2) can be approximated as follows:

(4) $$R\left(t_{m}\right)\approx R_{0}-\left(v_{t\_radical}-v_{a\_radical}\right)t_{m}$$

$v_{t\_radical}$ represents the radial speed of target, and $v_{a\_radical}$ represents the radial speed of airborne radar.

Equation (2) represents the range of the target as a Taylor series expansion, with higher-order terms ignored for simplicity. Equation (3) defines the relationship between the target’s radial velocity and the radar’s relative velocity, providing the basis for subsequent signal processing.

### 2.2. Signal Model

According to the working principle of the maritime surveillance radar, the radar transmits the LFM signals, while the sea surface target reflects echoes according to the radar cross-section (RCS). Consider a maritime surveillance radar on an aircraft emitting a series of pulses [19]:

(5) $$a\left(t\right)=p\left(t-kT_{i}\right)\exp\left[-j2\pi f_{c}\left(t-mT_{i}\right)\right]$$

where t represents the fast (range) time, m is integer, $f_{c}$ and $T_{i}$ denote the carrier frequency and the interpulse period, respectively. Typically, it is a chirp pulse, given by the following [19]:

(6) $$p\left(t\right)=rect\left(\frac{t}{T_{P}}\right)\exp\left(j\pi K_{r}t^{2}\right)$$

where $T_{P}$ and $K_{r}$ denote the pulse duration and the chirp rate, respectively. $rect\left(\cdot \right)$ is the rectangular window function [20,21,22,23]. Now, assume that this pulse train illuminates the target at a range R(t). The echoes from the sea surface target are recorded in a two-dimensional array $s\left(t,t_{m}\right)$ where $t_{m}=t-mT_{1}$ is known as the slow (azimuth) time. Then, in terms of these variables, the echoes after down-converting can yield the following:

(7) $$s\left(t,t_{m}\right)=\sigma \left(x,y\right)\cdot rect\left[\frac{t-R\left(t_{m}\right)}{T_{P}}\right]\exp\left\{j\pi K_{r}{\left[t-\frac{2R\left(t_{m}\right)}{c}\right]}^{2}\right\}\exp\left[-j\frac{4\pi R\left(t_{m}\right)}{\lambda }\right]$$

where σ(x,y) denotes the backscattering coefficient of the target, c represents the speed of light, and λ is the wavelength. Suppose that the backscattering coefficient of the target varies negligibly during the pulse interval $T_{p}$. We define the radial speed of the airborne radar relative to the target as $v_{radical}$. Substituting (4) into (7), we can obtain the following:

(8) $$\begin{array}{l} s\left(t,t_{m}\right)\\ =\sigma \left(x,y\right)\cdot rect\left[\frac{t-R_{0}+v_{radical}t_{m}}{T_{P}}\right]\exp\left\{j\pi K_{r}{\left[t-\frac{2\left(R_{0}-v_{radical}t_{m}\right)}{c}\right]}^{2}\right\}\exp\left[-j\frac{4\pi \left(R_{0}-v_{radical}t_{m}\right)}{\lambda }\right] \end{array}$$

where $v_{radical}=v_{t\_radical}-v_{a\_radical}$. Then, phase filtering along the azimuth direction is performed to (8) using the filter:

(9) $$h_{1}(t_{m})=\exp\left[+j\frac{4\pi \left(v_{a\_radical}t_{m}\right)}{\lambda }\right]$$

The filtered signal can be written as follows:

(10) $$\begin{array}{l} s_{1}\left(t,t_{m}\right)\\ =\sigma \left(x,y\right)\cdot rect\left[\frac{t-R_{0}+v_{radical}t_{m}}{T_{P}}\right]\exp\left\{j\pi K_{r}{\left[t-\frac{2\left(R_{0}-v_{radical}t_{m}\right)}{c}\right]}^{2}\right\}\exp\left[-j\frac{4\pi \left(R_{0}-v_{t\_radical}t_{m}\right)}{\lambda }\right] \end{array}$$

The Fourier transform of (10) over the fast time t yields the following:

(11) $$S_{1}\left(f,t_{m}\right)=\sigma \left(x,y\right)\cdot \exp\left(-j\pi \frac{f^{2}}{K_{r}}\right)\exp\left[-j\frac{4\pi \left(R_{0}-v_{radical}t_{m}\right)}{c}f\right]\exp\left[-j\frac{4\pi \left(R_{0}-v_{t\_radical}t_{m}\right)}{\lambda }\right]$$

As can be seen, the position of the adjacent pulse envelope changes at a speed of $v_{radical}$. Then, matched filtering along the range performed to (11) can be designed as follows [24,25]:

(12) $$H_{2}\left(f,t_{m}\right)=\exp\left(-j\frac{4\pi v_{a\_radical}t_{m}}{c}f\right)\exp\left(j\pi \frac{f^{2}}{K_{r}}\right)$$

Therefore, Formula (11) after filtering can be written as follows:

(13) $$S_{2}\left(f,t_{m}\right)=\sigma \left(x,y\right)\cdot \exp\left[-j\frac{4\pi \left(R_{0}-v_{t\_radical}t_{m}\right)}{c}f\right]\exp\left[-j\frac{4\pi \left(R_{0}-v_{t\_radical}t_{m}\right)}{\lambda }\right]$$

Performing an inverse Fourier transform to (13) along the fast time t yields [18] the following:

(14) $$s_{2}\left(t,t_{m}\right)=\sigma \left(x,y\right)\cdot T_{P}\cdot \sin c\left\{B\left[t-\frac{2\left(R_{0}-v_{t\_radical}t_{m}\right)}{c}\right]\right\}\exp\left[-j\frac{4\pi \left(R_{0}-v_{t\_radical}t_{m}\right)}{\lambda }\right]$$

Generally, for the stationary target and slow-moving target, the speed of the sea surface target is too small. The influence of the target speed on the position of each pulse envelope can be ignored. The resultant range migration should be far less than a pixel spacing. Thus, the above formula approach to [26] is as follows:

(15) $$s_{2}\left(t,t_{m}\right)\approx \sigma \left(x,y\right)\cdot T_{P}\cdot \sin c\left[B\left(t-\frac{2R_{0}}{c}\right)\right]\exp\left[-j\frac{4\pi \left(R_{0}-v_{t\_radical}t_{m}\right)}{\lambda }\right]$$

where B denotes the emitting signal bandwidth. The Fourier transform of (15) over the slow time $t_{m}$ can be expressed as follows:

(16) $$S_{out}\left(t,f_{m}\right)\approx \sigma \left(x,y\right)\cdot T_{P}\cdot \sin c\left[B\left(t-\frac{2R_{0}}{c}\right)\right]\cdot \sin c\left[B_{m}\left(f_{m}-\frac{2v_{t\_radical}}{\lambda }\right)\right]\exp\left(-j\frac{4\pi R_{0}}{\lambda }\right)$$

where $B_{m}$ is the Doppler bandwidth. As can be seen, the echo from the stationary target and slow-moving target can be accumulated along the azimuth.

### 2.3. Tracking Result Analysis

Equation (14) indicates that the radical speed of the sea surface target affects the position of the signal envelope after range compression. The velocity migration due to the airborne radar is completely compensated, while the one due to the sea surface target is preserved. Especially when the position deviations of the pulse series are beyond one pixel along the azimuth during the pulse accumulation period, a defocusing effect on the output signal is caused.

**Figure 2:**
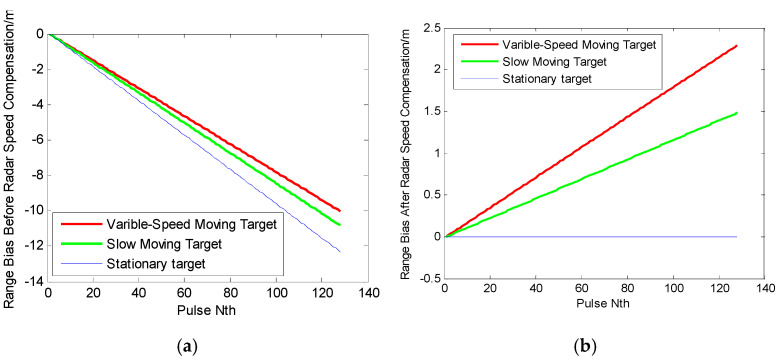
Comparison between the range bias before and after radar speed compensation: (**a**) range bias before radar speed compensation; and (**b**) range bias after radar speed compensation.

As an example, targets with three different motion states have been simulated, utilizing the parameters listed in Table 1. The velocity migration of the range compressed pulse during the pulse accumulation period is shown in Figure 2. The red, green, and blue curves, respectively, represent the range biases due to the variable-speed target, the slow-moving target, and the stationary target. Figure 2a,b present the range bias before and after the radar speed compensation, respectively. Clearly, the range biases of three targets change with the increase in pulse number. Note that such a range bias of the stationary target is completely compensated in Figure 2b, while those for the variable-speed target and slow-moving target are partially retained. Figure 2b illustrates that the variable-speed target induces greater range biases than the slow-moving one.

In the simulation experiments, three types of targets with different motion states are modeled: stationary targets with zero velocity and acceleration, slow-moving targets modeled as uniformly accelerated motion with initial velocity *a* and acceleration *b*, and variable-speed targets with initial velocity *c* and acceleration *d*. These motion forms are illustrated in Table 2 and Figure 2.

**Figure 3:**
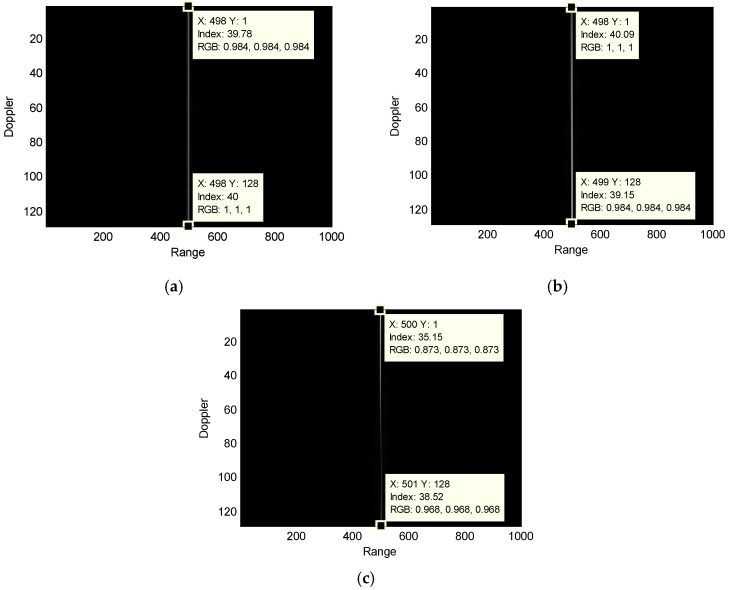
Comparison among the compressed signals of target with only radar speed compensation: (**a**) stationary target; (**b**) slow-moving target; and (**c**) variable-speed target.

To further analyze the impact of the motion state on the performance of the traditional algorithm, an airborne maritime surveillance radar geometry, as shown in Figure 1, is considered. The airborne radar parameters are listed in Table 1 and those for the targets are listed in Table 2. The target is placed at the center of the detecting scene. The airborne maritime surveillance radar transmits and receives an echo from the target simultaneously. Figure 3 presents the range-compressed signals of the target after radar speed compensation. As can be seen, the pulses due to the stationary target are located at the same range unit, while those for the moving targets show a varying position deviation along the range. Especially, the range position deviation due to the variable-speed target is larger than that of slow-moving target.

**Figure 4:**
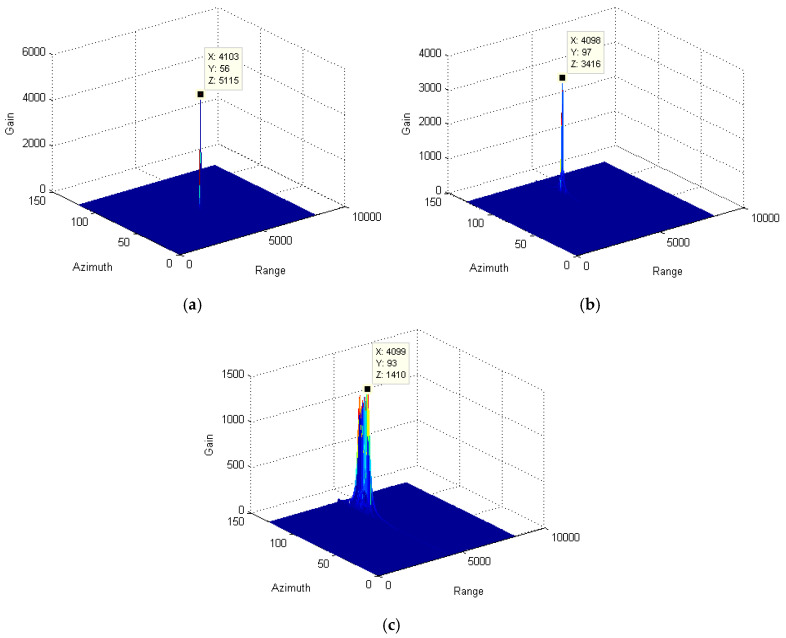
Comparison among the accumulated signals of target using the traditional method: (**a**) stationary target; (**b**) slow-moving target; and (**c**) variable-speed target.

Through the azimuth pulse accumulation, the resultant signals are obtained and depicted in Figure 4. Since the range migration and azimuth phase due to the relative speed between the target and radar are completely compensated for with the stationary target, the maximum accumulation gain is obtained, as shown in Figure 4a. However, for the moving targets, the radar speed compensation cannot eliminate the impact of the radical speed of the target. The residual range migration and azimuth phase decrease the signal gain significantly. By comparing Figure 4b,c, the gain of the variable-speed target is much less than that of the slow-moving target. Therefore, when utilizing the traditional method to track the variable-speed target, the defocusing effect is more prominent. It will further affect the detection and recognition of the target in low-signal-to-noise-ratio environments.

## 3. Improved Tracking Algorithm of Maritime Moving Targets

### 3.1. Proposed Tracking Scheme

According to the principle of the traditional method introduced in Section 2, we have the following findings: the traditional method can be applied for tracking those slowly moving targets. However, for the variable-speed target, the state of motion will be unpredictable within a short duration. It will result in envelope misalignment, produce a nonlinear phase error, and reduce the focusing performance.

**Figure 5:**
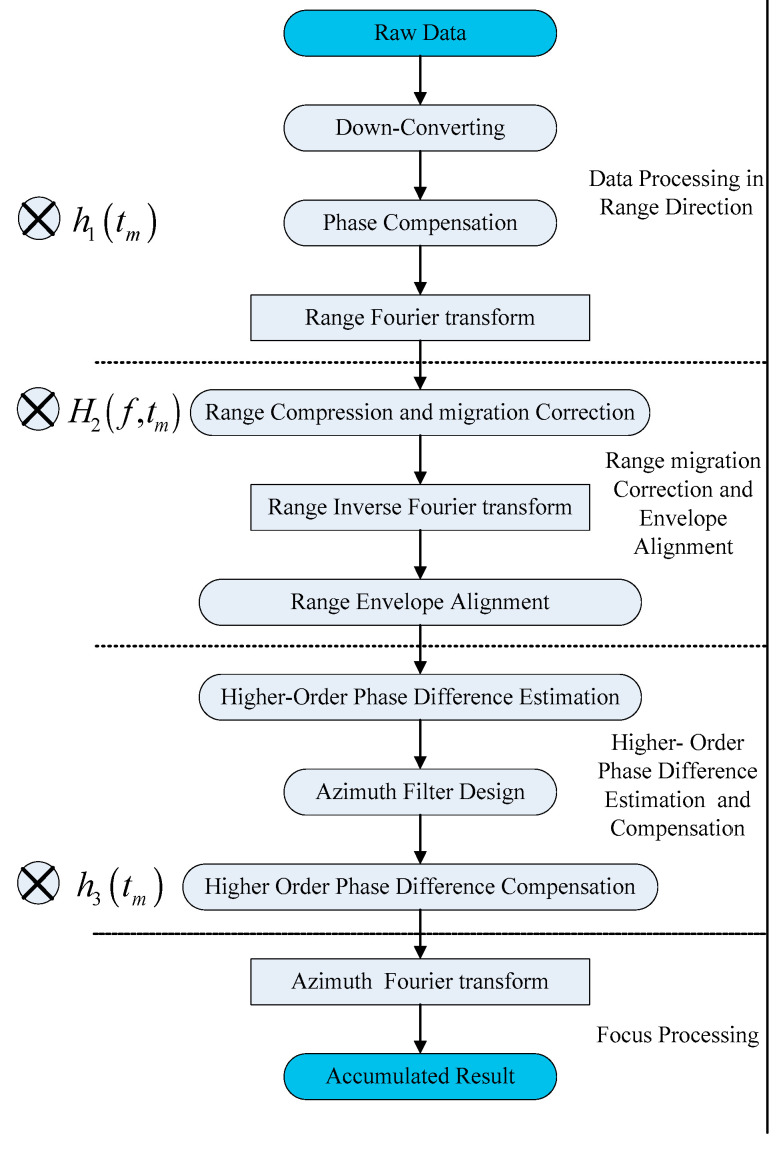
Detailed steps of the improved tracking algorithm.

The improved tracking algorithm achieves range envelope alignment through envelope correlation. The azimuth higher-order phase error due to the motion of targets is estimated and compensated subsequently. As shown in Figure 5, the detailed process of our algorithm includes four steps as follows:

1. Data processing in the range direction. The echo data after down-converting are recorded in a two-dimensional array. Such a signal is composed of two parts: the delay LFM signals and the phase term due to the Doppler changes. This step is to compensate the azimuth phase due to the motion of the radar platform. Then, Fourier transform is performed along the range direction.
2. Range migration correction and envelope alignment. In this step, the pulse compression and migration correction along the range direction is implemented through the matched filtering and radical speed of radar compensation. After the range inverse Fourier transform, the unpredictable range migration due to the motion of the target is corrected through envelope alignment.
3. Higher-order phase difference estimation and compensation. This step initially calculates the phase gradient between the adjacent pulses along the azimuth. Meanwhile, the higher-order phase difference is estimated through low-frequency filtering. Then, the resultant azimuth filter is utilized to compensate such a higher-order phase.
4. Focusing processing. After the above three steps, the signal data can be considered as a range-aligned pulse series with a continuous linear phase. Such pulse series are accumulated through azimuth Fourier transform.

### 3.2. Range Envelope Alignment

Generally, since the maritime surveillance radar receives echoes from those slowly moving targets within a short duration, the impact of the target motion on range migration can be negligible. However, for variable-speed targets, the higher velocity of the target will induce unpredictable range migration. Such range migration exceeding a pixel limit will seriously affect the signal focusing. Therefore, the range envelope alignment, as one of the key steps for signal focusing processing, should be considered in this part.

Formula (14) shows the range-compressed signal of the mth pulse after radar radical speed compensation, while ignoring the higher-order term in the Taylor series expansion of $R\left(t_{m}\right)$. Consider the higher-order term induced by the target motion; Formula (14) can be written as follows [27]:

(17) $$\begin{array}{l} s_{2}\left(t,t_{m}\right)\\ =\sigma \left(x,y\right)\cdot T_{P}\cdot \sin c\left\{B\left[t-\frac{2\left(R_{0}-v_{t\_radical}t_{m}+o\left({t_{m}}^{2}\right)\right)}{c}\right]\right\}\exp\left\{-j\frac{4\pi \left[R_{0}-v_{t\_radical}t_{m}+o\left({t_{m}}^{2}\right)\right]}{\lambda }\right\} \end{array}$$

Similarly, the (m+1)th pulse can be written as follows [27]:

(18) $$\begin{array}{l} s_{2}\left(t,t_{m+1}\right)\\ =\sigma \left(x,y\right)\cdot T_{P}\cdot \sin c\left\{B\left[t-\frac{2\left(R_{0}-v_{t\_radical}t_{m+1}+o\left({t_{m+1}}^{2}\right)\right)}{c}\right]\right\}\exp\left\{-j\frac{4\pi \left[R_{0}-v_{t\_radical}t_{m+1}+o\left({t_{m+1}}^{2}\right)\right]}{\lambda }\right\}\; \end{array}$$

Combining (17) and (18), the relationship between adjacent pulses can be derived as follows:

(19) $$s_{2}\left(t,t_{m+1}\right)=s_{2}\left\{t+\frac{2\left[v_{t\_radical}\Delta T+o\left(\Delta T^{2}\right)\right]}{c},t_{m}\right\}\exp\left\{j\frac{4\pi \left[v_{t\_radical}\Delta T+o\left(\Delta T^{2}\right)\right]}{\lambda }\right\}$$

where ΔT denotes the pulse repetition period. $o\left(\Delta T^{2}\right)$, as the higher order of ΔT, shows the unpredictable motion of the target relative to the airborne radar. We define $\Delta r=v_{t\_radical}\Delta T$. Moreover, Formula (19) indicates a perfect envelope correlation between the adjacent pulses. The envelope correlation can be used to calculate the position deviation Δr between the adjacent pulses.

Based on the above analysis, we propose a novel envelope correlation algorithm to achieve range envelope alignment. Correlation, a measure of similarity between two adjacent pulses, is mainly affected by the properties of the reflected echoes and several system parameters [28]. The envelope correlation coefficient γ between the adjacent pulses is defined as follows:

(20) $$\gamma =\frac{\int \left|s_{2}\left(t,t_{m}\right)\right|\left|s_{2}\left(t,t_{m+1}\right)\right|dt}{\sqrt{\int {\left|s_{2}\left(t,t_{m}\right)\right|}^{2}dt\int {\left|s_{2}\left(t,t_{m+1}\right)\right|}^{2}dt}}$$

Substituting (19) into (20), we can obtain the following:

(21) $$\gamma =\frac{\int \left|s_{2}\left(t,t_{m}\right)\right|\left|s_{2}\left\{t+\frac{2\left[\Delta r+o\left(\Delta T^{2}\right)\right]}{c},t_{m}\right\}\right|dt}{\sqrt{\int {\left|s_{2}\left(t,t_{m}\right)\right|}^{2}dt\int {\left|s_{2}\left\{t+\frac{2\left[\Delta r+o\left(\Delta T^{2}\right)\right]}{c},t_{m}\right\}\right|}^{2}dt}}$$

As can be seen, for $\Delta r+o\left(\Delta T^{2}\right)=0$ in Equation (20), γ approaches the maximum. Therefore, the correlation calculation is the best approach for estimating the position deviation between the adjacent pulses. In practice, to obtain a more precise $\Delta r+o\left(\Delta T^{2}\right)$, the range envelope interpolation is implemented before the correlation calculation. Then, the position deviation between the adjacent pulses is compensated by using $\Delta r+o\left(\Delta T^{2}\right)$. Therefore, Formula (17) is rearranged as follows:

(22) $$s_{2}\left(t,t_{m}\right)=\sigma \left(x,y\right)\cdot T_{P}\cdot \sin c\left\{B\left[t-\frac{2R_{0}}{c}\right]\right\}\exp\left\{-j\frac{4\pi \left[R_{0}-v_{t\_radical}t_{m}+o\left({t_{m}}^{2}\right)\right]}{\lambda }\right\}$$

Clearly, the range migration induced by the radical speed of the target is completely compensated through envelope alignment.

### 3.3. Azimuth Phase Filtering

Initially, to obtain the phase difference between the adjacent pulses, complex conjugate multiplication is implemented. Therefore, the phase difference of such adjacent pulses can be obtained as follows:

(23) $$\Delta \left(\varphi_{m}\right)=\frac{\lambda }{4\pi }\cdot Arg\left[s_{2}{\left(t,t_{m}\right)}^{*}\cdot s_{2}\left(t,t_{m+1}\right)\right]$$

Substituting (22) into (23), the phase difference between two adjacent pulses yields the following:

(24) $$\Delta \left(\varphi_{m}\right)=v_{t\_radical}\Delta T-o\left(\Delta T^{2}\right)$$

Obviously, the quality of such a phase difference is mainly limited by the unpredictable motion of the target relative to $o\left(\Delta T^{2}\right)$.

To obtain the higher-order phase difference caused by the target, low-frequency filtering is implemented along the azimuth direction [29,30,31,32]. Initially, the low frequency in each filtering window due to the main motion feature of the target is removed from the original phase difference. Subsequently, the residual phase part $o\left(\Delta T^{2}\right)$ is estimated. And it is compensated by utilizing the phase filter:

(25) $$h_{3}\left(t_{m}\right)=\exp\left[j\frac{4\pi }{\lambda }o\left({t_{m}}^{2}\right)\right]$$

Formula (22) after filtering shows the continuous linear phase along the azimuth direction. Therefore, the above filtering procedure removes most of the high-order phase while still preserving the low-frequency phase well. The resultant signal, as shown in Formula (15), will be precisely focused after the azimuth Fourier transform.

## 4. Experimental Analysis

The aforementioned sections have analyzed the impact of the target motion on signal focusing and addressed the procedures of our algorithm, together with a detailed theoretical analysis. In what follows, to validate the robustness of our algorithm for tracking the target, the simulation results are provided based on the airborne maritime surveillance radar geometry. The performance of envelope alignment and azimuth phase filtering are, respectively, analyzed.

### 4.1. Envelope Alignment Performance Analysis

Envelope alignment is utilized to reduce the effect of the radical speed of the target on velocity migration and correct the range deviation of signal envelopes. According to Formulae (21) and (22), such a position deviation between the adjacent pulses is derived through correlation and automatically compensated.

**Figure 6:**
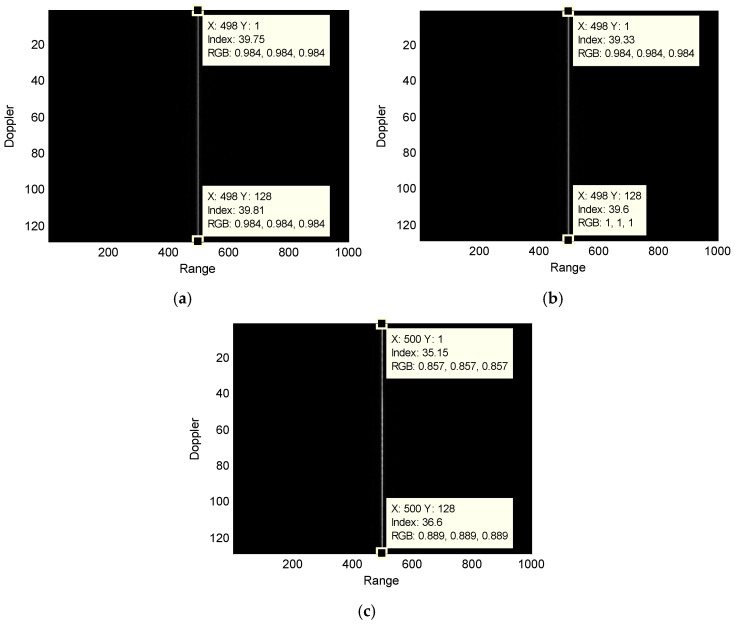
Comparison among the compressed signals of target after envelope alignment: (**a**) stationary target; (**b**) slow-moving target; and (**c**) variable-speed target.

Figure 6 presents the envelope-aligned signals from three targets with different moving speeds. By comparing Figure 3 and Figure 6, the residual range migrations for moving targets are completely compensated according to the position of the first pulse envelope. For the stationary target, range envelope alignment cannot change the position of the pulse. Clearly, range envelope alignment can effectively compensate the residual range migrations caused by the uncertain movement of targets.

**Figure 7:**
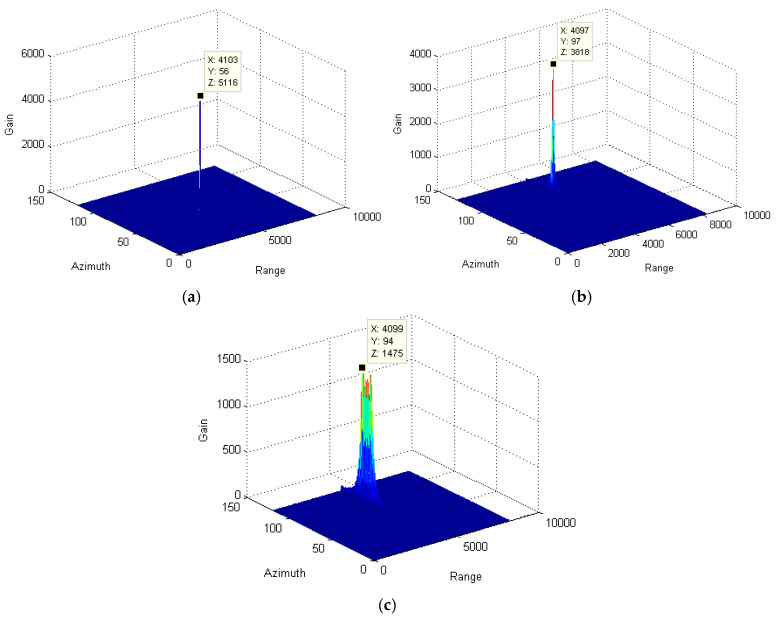
Comparison among the accumulated signals of target with only envelope alignment: (**a**) stationary target; (**b**) slow-moving target; and (**c**) variable-speed target.

To further analyze the effect of envelope alignment on signal focusing, the range-aligned signals are accumulated through the azimuth Fourier transform. The resultant signals are shown in Figure 6. By comparing Figure 4 and Figure 6, the accumulating gain of the stationary target, slow-moving target, and varying-speed target, respectively, increases to 0.001 dB, 0.97 dB, and 0.39 dB. Clearly, the range envelope alignment is more effective for the moving target than the stationary one. However, due to the high-order phase part caused by the radical speed of the target, the focusing effectiveness for moving targets is limited, as shown in Figure 7b,c. Therefore, to derive a perfect focusing result, phase filtering along the azimuth direction is essential in order to eliminate the residual phase deviation.

The experimental results demonstrate that the proposed algorithm achieves significant signal accumulation gains: 0.005 dB for stationary targets, 2.6 dB for slow-moving targets, and 9.6 dB for variable-speed targets. These results highlight the algorithm’s effectiveness, particularly for variable-speed targets.

### 4.2. Azimuth Phase Filtering Result Analysis

Consider the neglected term $o\left({t_{m}}^{2}\right)$ in Formula (4), Formula (15) can be written as follows:

(26) $$s_{2}\left(t,t_{m}\right)\approx \sigma \left(x,y\right)\cdot T_{P}\cdot \sin c\left[B\left(t-\frac{2R_{0}}{c}\right)\right]\exp\left[-j\frac{4\pi \left(R_{0}-v_{t\_radical}t_{m}+o\left({t_{m}}^{2}\right)\right)}{\lambda }\right]$$

**Figure 8:**
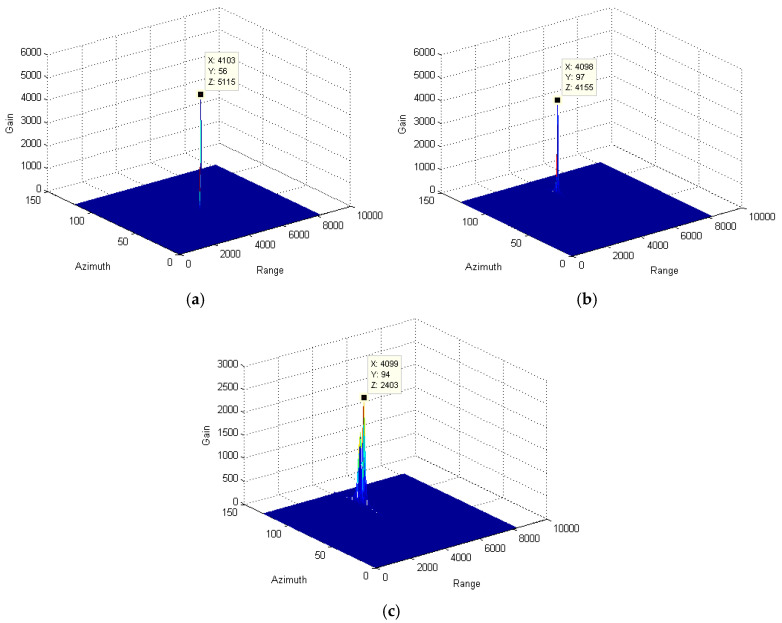
Comparison among the accumulated signals of target with only azimuth phase filtering. (**a**) stationary target; (**b**) slow-moving target; and (**c**) variable-speed target.

In the above formula, the residual phase part of $o\left({t_{m}}^{2}\right)$ due to the radical speed of the target will further affect the target position and signal focusing along the azimuth direction. Especially for those varying-speed targets, the residual phase part seriously decreases the signal-accumulating gain and results in signal defocusing. To effectively solve this problem, an azimuth phase filter is utilized to reduce the high-order phase error. For comparison purposes, signal-accumulating results from targets with a different moving state is presented, as shown in Figure 8. By comparing Figure 4c and Figure 8c, the signal-accumulating gains of the varying-speed target and slow-moving target are significantly improved. However, for the stationary target, since the high-order phase error can be ignored, the effectiveness of our method is limited.

**Figure 9:**
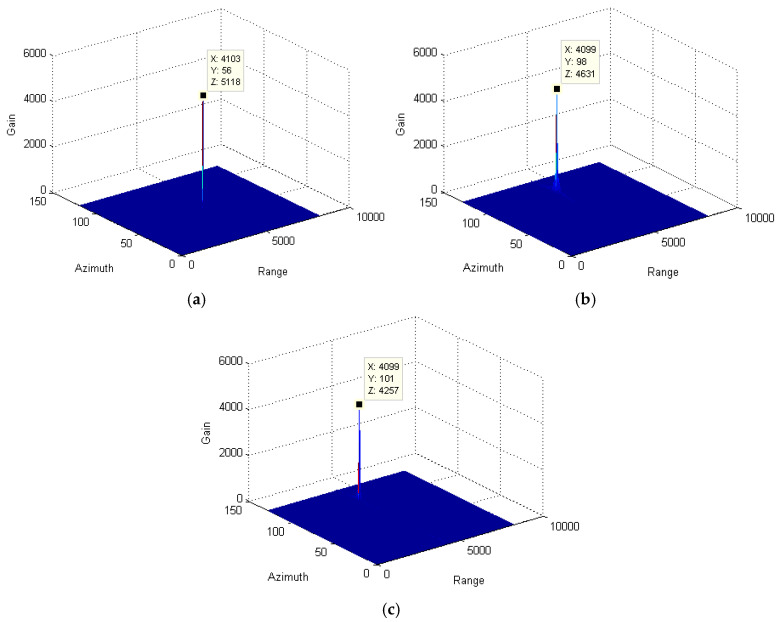
Comparison among the accumulated signals of target using our method: (**a**) stationary target; (**b**) slow-moving target; and (**c**) variable-speed target.

In this part, envelope alignment is initially utilized to correct the range deviation of envelopes after range compression and radar speed compensation. Subsequently, the azimuth phase filter is used to remove the residual high-order phase while still preserving the low-frequency phase well. Finally, the accumulated signal is obtained, as shown in Figure 9. By comparing Figure 4 and Figure 9, we obtain that the accumulating gain of the stationary target, slow-moving target, and varying-speed target, respectively, increases to 0.005 dB, 2.6 dB, and 9.6 dB. Clearly, the focusing effectiveness for the signal from the varying-speed target is significantly improved, as shown in Figure 8c. Taking full advantage of the envelope alignment and azimuth phase filter, the residual migration and high-order phase are greatly reduced. It again demonstrates the good performance of the proposed algorithm.

## 5. Conclusions

In this work, the moving effect of the non-cooperating target on airborne radar tracking has been analyzed and a novel algorithm jointly employing envelope alignment and the azimuth phase filter has been proposed. Moreover, the following three major findings have been obtained:

(1) Range envelope alignment can effectively extract the position deviations between the adjacent pulses and precisely correct the unpredictable velocity migration induced by the radical speed of the target. Such position deviations after envelope alignment are narrowed within one range’s pixel space. The simulation results demonstrate that the proposed algorithm significantly improves the signal-focusing performance, achieving a signal accumulation gain of 9.6 dB for variable-speed targets.
(2) The azimuth phase filter removes most of the high-order phase deviation while still preserving the low-frequency phase well. The resultant signals after pulse accumulation shows good focusing performance.
(3) The primary innovation of the proposed algorithm lies in its ability to compensate for the motion of both the target and the platform, which significantly enhances the signal focusing and tracking accuracy compared to traditional methods that only compensate for platform motion.

As also shown by the simulation results based on the airborne maritime surveillance radar geometry, the proposed algorithm combining range envelope alignment and the azimuth phase filter can effectively track targets with different motion states. Moreover, the tracking results also provide a good starting point for possible sea surface target identification.

## Figures and Tables

**Table 1:** Simulation parameters of airborne radar.

Parameter	Value
Carrier frequency	9.6 GHz
Pulse duration	200 μs
Pulse repetition frequency	1000 Hz
Flight velocity along y-axis	50 m/s
Flight altitude	3000 m
Signal bandwidth	80 MHz
Sampling frequency	120 MHz
Accumulated pulse number	128

**Table 2:** Motion state parameters of target.

Parameter	Value
Initial position of target	[0, 0, 0] m
Velocity of stationary target	[0, 0, 0] m/s
Velocity of slow-moving target	[10, 0, 10] m/s
Initial velocity of variable-speed target	[13, 0, 28] m/s

## Data Availability

Data are contained within the article.
